# A computational approach to selective attention in embodied approaches to cognitive archaeology

**DOI:** 10.1098/rsif.2024.0508

**Published:** 2024-10-09

**Authors:** Axel Constant, Laura Desirèe Di Paolo, Avel Guénin-Carlut, Luis M. Martinez, Felipe Criado-Boado, Johannes Müeller, Andy Clark

**Affiliations:** ^1^ School of Engineering and Informatics, University of Sussex, Falmer (Brighton & Hove), UK; ^2^ Developmental Psychology, ChatLab, University of Sussex, Falmer (Brighton & Hove), UK; ^3^ Instituto de Neurociencias de Alicante, Alicante, Spain; ^4^ Instituto de Ciencias del Patrimonio, Santiago de Compostela, Galicia, Spain; ^5^ Kiel University, Kiel, Germany

**Keywords:** computational cognitive archaeology, computational cognitive science, hidden-Markov models, predictive processing, attention

## Abstract

This article proposes a novel computational approach to embodied approaches in cognitive archaeology called computational cognitive archaeology (CCA). We argue that cognitive archaeology, understood as the study of the human mind based on archaeological findings such as artefacts and material remains excavated and interpreted in the present, can benefit from the integration of novel methods in computational neuroscience interested in modelling the way the brain, the body and the environment are coupled and parameterized to allow for adaptive behaviour. We discuss the kind of tasks that CCA may engage in with a narrative example of how one can model the cumulative cultural evolution of the material and cognitive components of technologies, focusing on the case of knapping technology. This article thus provides a novel theoretical framework to formalize research in cognitive archaeology using recent developments in computational neuroscience.

## 1. Introduction

Attention is a powerful tool in an animal’s mental repertoire. By attending to certain aspects of unfolding scenes, often complex scenes, animals make the most of various limited resources, effectively optimizing the outlay of time, processing and energy as we perform our tasks and approach our goals. But there is another, far less-studied side to this familiar process. Our own environments. Artefacts and behaviour distinctive of humankind direct and manage attention. The drive of attention by environmental structures thus appears as a major bridge linking cognition and the production and use of material artefacts.

Attention comes in many varieties and plays multiple cognitive roles [1]. ‘Focal attention’ occurs when the brain is oriented closely towards some small regions, points or features of the environment. Attention can also be ‘distributed’ more widely across a scene, or ‘divided’ among different tasks. It can also be directed purposefully or ‘captured’ by some strong or salient external stimuli, such as a loud noise. But despite this surface variety, there is a common core to all the phenomena grouped under the banner of attention. That common core involves the role of attention as a cognitive tool amplifying the effects of some types or bodies of information at the temporary expense of others.

Attending, as we shall use the term, is the process whereby the use of limited cognitive resources is optimized to explore and learn the structure of the world efficiently [2]. We attend to sources of relevant sensory information that allow us to form accurate knowledge, beliefs or expectations about world states (e.g. attending to your watch to know the time so that you can arrive on time for your appointment). This is done either by the covert selection of sensory information, achieved by increasing the internal influence of select aspects of the sensory signal [3,4] or through overt action, selectively harvesting sensory data needed to improve our knowledge of the world [5,6]. These correspond, for example, to turning your watch face towards you (overt action) and to increasing the internal influence of the sensory information that is then made available.

In addition, artefacts themselves (e.g. your watch) play a key role in supporting the processing of information [7,8], either by making relevant sensory signals stand out, or by channelling information and pre-processing its relevance (e.g. the shape of a glass affording its graspability). Thus, the structured world that we both build and encounter, plays a powerful role in directing and channelling attention and (when all goes well) facilitates the job of the neuronal mechanisms [9] in supporting successful behaviours.

In the treatment that follows, we show how recent neuroscientific approaches to selective attention can be generalized to offer a computational interpretation of the attentional role of material objects. In particular, we show how recent neurocomputational approaches to thinking and attending could offer both a vocabulary and a framework for thinking about some of the ways material and social innovations do double-duty as mental innovations. We position our work as a contribution to the broad field of cognitive archaeology—the study of the human mind based on archaeological findings such as artefacts and material remains excavated and interpreted in the present—and computational neuroscience. The goal of this article is to provide a proof of concept for the application of the proposed computational framework to some issues in cognitive archaeology, namely, issues related to how cognitive archaeologists—especially those interested in embodied, embedded and enactive approaches to cognition—perform inference about cognitive capacities of humans of the past such as selective attention based on the analysis of material artefacts.

We shall refer to our approach as computational cognitive archaeology (CCA), which in the present treatment is applied to attention understood as an embodied and materially scaffolded phenomenon. Given the interdisciplinary nature of our proposal, there will naturally be a vast literature with many different positions and arguments that could have been addressed and debated. For instance, studies on the integration of attention and visuospatial processing in the context of toolmaking could have been discussed within the context of our approach [10–12], and we could have decided to take a deep dive into the fundamental issues that relate 4E (embodied, embedded, enacted, extended) cognition (for a review see [13]) and ecological psychology [14] with the evolution of cognitive functions. We believe that our approach does not directly contradict the positions in the above work for it makes no ontological commitment to what should count as mind and how one should envisage the connection between mind and materiality. Our approach simply provides computational tools that may be useful to reflect on the broad claim that change in cognition should be studied by considering change in materiality, and attempts to provide tooling to advance in that direction.

Section 2 reviews central concepts in the cognitive archaeology of attention in relation to embodied cognition and cumulative cultural evolution. Section 3 discusses some recent developments in the computational neurobiology of attention, which will be reused in §4 onwards to support our proposed model of attention, specifically within the context of knapping, the practice of ‘striking a stone with another stone or something softer than a stone, such as bone or wood’ [15]. Section 4 provides a tentative definition of CCA; one method derived from which is then applied to study of the co-evolution of attention and knapping technology in §5.

## 2. The cognitive archaeology of attention

### 2.1. Cognitive archaeology and embodied cognition

Cognitive archaeology is sometimes situated within embodied, embedded and enacted theories of cognition [16]. In those theories, both cognitive functions (e.g. action, perception and learning), and the content of mental experiences, are scaffolded within sensory–motor interactions with the physical and material world [17,18]. Accordingly, the starting assumption of contemporary approaches to cognitive archaeology focusing on embodied, embedded and enacted theories of cognition is that (what we call) the mind, and its functions, are in many ways the result of our engagements with material culture, rather than simply its cause [19–23]. In the strongest versions of this idea, the mind itself is said to extend beyond the whirring and grinding of the inner neuronal mechanisms, and to encompass some of what might more commonly be seen as the environment.

Cognitive extensions occur, it has been argued, when inner activity, in order to reach our goals, becomes routinely dependent on specific cultural settings, and material props and aids [24–26]. In such cases, cognition ‘extends beyond the skull’ [16,24] into constitutive embodied engagements with the world, as artefacts and social institutions [27]. The material world, while bringing information about its structures, also ‘affords’ action possibilities [28,29]. The world allows the unfolding of the perceptual experience, promoted by the affordances of the material objects and spaces (e.g. the cane of the blind man extends the man’s perception through the reachability the cane affords [30]). This suggests that minds and the material world are intertwined [31]; the mind leveraging the world through action–perception loops and the world functioning as a repository of action-possibilities scaffolding cognition.

Epistemologically, this licenses cognitive archaeologists to make inferences about past minds on the basis of archaeological records, as those records are a kind of ‘fossil trail’ not just of the effects of previous modes of thinking (the ‘grist’), but of parts of the thinking machinery itself (the ‘mill’) [32]. This means that, amongst other things, artefacts are a window onto changing patterns of attending [33]—changing patterns whose importance for the emergence of the modern mind is only recently starting to come into view [34].

### 2.2. Cognitive archaeology and cumulative cultural evolution

Cumulative cultural evolution is a process by which we expand on and build upon inventions (processes, artefacts, industries and technologies) made by others [35], thereby allowing for the exploitation of different forms of transmission and learning strategies. Cumulative cultural evolution is a process of cultural transmission, or passing onto future generations of learning that exceeds what could be passed and learned by any single individual alone. It is a complex process that combines various forms of material inheritance [36]. Some cognitive archaeologists observe this process through the lens of embodied cognition, and therefore, according to the embodied view, artefacts become the field for inferring features about human minds of the past. If cognition is indeed embodied, and the problem of inference about past minds can indeed be framed within a cultural-evolutionary framework [37], one can assume that the mind co-evolves to shape human social cognition and institutions [38,39] and to shape technologies themselves (for a definition of technologies, see below, §5.1 and box 1).

Box 1. **Attention and salience**

Computationally, the susceptibility of an update in Bayesian beliefs about the conditional relation between a sensation and its possible causes depends on the precision of that belief (usually modelled as the inverse covariance of that belief, which itself corresponds to a likelihood mapping between world states and sensory observations) [40]. Covert attention is the process whereby one modulates the precision afforded to sensory evidence. This is known as the precision ‘weighting’ of the evidence and is thought to be realized by the synaptic modulation of neurotransmitters such as dopamine and acetylcholine in the visual cortex [41]. Overt attention, in turn, is the process of selectively attending to specific external stimuli through action.

Attentional salience is the measure of how much new sensory information a given action is expected to bring. Attentional salience, in turn, rests on the active seeking of novel sensory data needed to construct percepts [42,43]. Hence, salience is an attribute of an action, and not of a model parameter, as is the case with covert attention understood as precision. Salient actions are selected relative to some measure of how a change in Bayesian beliefs would ensue after having sampled the novel data. At the scale of ontogeny, and for individual organisms, action that brings about known sensory data has no salience, as no new knowledge is acquired through their performance (e.g. there is little need to watch the time once on your phone and once on your watch) [40]. The same applies if there is a great uncertainty over beliefs about the relation between data and causes to be elucidated (e.g. if I think that my watch is malfunctioning, there is little use in looking at it). Hence, formally, the salience of an action refers to the extent to which one believes an action will bring about information, or revise one’s beliefs about future states of the world—e.g. looking at the watch to resolve uncertainty in beliefs about the time of the day [44], which reduces uncertainty over beliefs about the sensory structure of the world.

Together, covert and overt information processing strategies have been described, computationally and neurobiologically, as belonging to the two distinct phenomena of attention and salience, respectively [45,46]. These can be used to explain the phenomenon of selective attention. Selective attention is the process whereby one focuses on sensory inputs deemed relevant while suppressing other sensory inputs deemed irrelevant. When looking at the watch, competing auditory information is momentarily dampened, but when listening to a flight announcement, auditory information is effectively prioritized. Competition in the sensory domain can be elicited exogenously (stimulus driven) and resolved through overt attentional processes (e.g. moving your head to look at the watch) selective samples through action), or it can be elicited endogenously, for instance, as one is dealing with competing thoughts [47], and can resolve through covert processes such as gain control—the process of changing the weight attributed to sensory data during perception. These attentional mechanisms work cooperatively, increasing the influence of (in this case) the visual information made available by the movement of the head and eyes.

Exogenous, stimulus driven and endogenous mechanisms of selective attention are coupled in the following way. Sensory attenuation through gain control optimizes the processing of sensory samples to form percepts and, by the same token, determine which future sensation would be needed for improving perceptual processing (i.e. determines the salience of action). In turn, salient actions (e.g. directed saccadic eye movement) are performed to select those sensations [48].

The fact that selective attention is ‘exogenously driven’ suggests that selective attention could be one such co-evolved cognitive function. Structures of the world invite the performance of certain actions that have proven adaptive in the past, in both human and non-human animals (e.g. socially transmitted foraging traditions, such as crack-opening nuts in capuchin monkeys [49–51]). In cognitive archaeology, selective attention in humans is explained by the co-evolution of visual responses to stimuli, such as visual ‘foraging’ tendencies with artefacts’ complexity, which would have in turn co-evolved with social complexity [34]. In particular, Criado-Boado *et al*. [34] have shown that visual foraging styles (i.e. exploratory patterns), investigated through the adoption of eye-tracking techniques, are determined by the artefacts’ material styles (i.e. shapes and spatial distribution of visual features), since changes in visual foraging styles correlate with changes in sets of ceramics, organized in chrono-cultural periods, which themselves follow the evolution of social institutions. Criado-Boado *et al*. thus bring strong evidence for arguing both that cognitive transformations are tied to artefactual and institutional social transformations, and that it is possible to track cognitive transformations through the tracking of transformations in the archaeological record.

Based on such behavioural studies and on theories in computational neurobiology, we suggest that it is possible to begin the exploration of the mechanisms that may underwrite the manner in which artefacts have driven change in selective attention.

## 3. The computational neurobiology of attention

### 3.1. Concepts of attention in cognitive neuroscience

Recent work in cognitive neuroscience known as active inference (ActInf) depicts the brain as an organ of prediction [45]. This work suggests perception, cognition and action as manifestations of a core computational strategy whose guiding principle is the long-term minimization of errors in prediction. Prediction errors can be corrected either by altering sensory predictions (perception, reason) or altering inputs (sensory sampling, action). At the heart of both these processes lies a knowledge base model called the ‘generative model’ that enables moment-by-moment predictions and input selection. The behaviour of the brain and cognition can be modelled as an optimization process of such a generative model [52,53]. For instance, a generative model can represent the joint probability of world states and sensations (e.g. locations in a visual scene, and the colour associated with each patch). In that case, brain behaviour underwriting perception (e.g. forming a percept of the current location and its associated colour in a visual scene) would be modelled as an inference over such a generative model, and action (e.g. inferring the next location in the visual scene towards which will be the next stop in my scan path) would be modelled as an inference over how states causing sensations will change over time. Inference over the generative model is realized through downward-flowing predictions about the shape of the current sensory signal that can be compared with the incoming signal. When it all matches up, we perceive (and understand) our worlds. But when mismatch occurs, prediction errors result. These carry information about any residual differences and enable the system to seek out a better guess—or else to act on the world, altering the inputs to fit the predictions. Perception and action thus co-emerge from an ongoing dance between predictions and prediction errors.

Importantly (and crucially, for our purposes), this dance is orchestrated by an additional key player: attention. Attention is the process that optimizes the so-called ‘precision-weighting’ on predictions and prediction errors, altering neural and bodily responses in ways that (as noted earlier) make the most of limited resources, deploying them in ways appropriate to both task and context. Attention and salience are brought together into one coherent framework, as distinct, but key mechanisms of information processing (see box 1). Attention is the process whereby gain control, or precision of beliefs about the world is adjusted to the changes of the weight attributed to relevant sensory data during perception. Salience is, in turn, an attribute of actions (such as saccadic eye movements) driving the selective sampling of the data deemed relevant for improving the knowledge of the world [40,46]. This view is complementary to the view of salience as a ‘saliency map’. It is suggested that salience corresponds to a saliency map that functions as a representation of the environment, or causes of sensations and that weighs sensory inputs according to its behavioural relevance and degree of contrast with respect to other inputs [54]. Under the definition of salience used here, the weight given to sensory inputs in the map, sensory beliefs or perceptual knowledge of the world are those determining the value of an action; the better at improving contrast the action will be (e.g. by bringing about evidence to increase the weight of a cell of the map against another cell), the more ‘salient’ the action will be. In short, here, attention refers to the internal revision of beliefs, and salience, or ‘relevance’ of the action refers to the extent to which a certain action is thought to improve beliefs revision by improving contrast in the saliency map.

For example, one of us recently had the experience of searching unsuccessfully for their car keys on a crowded work surface. The action of searching for the keys on that surface can be said to have salience in so far as a successful search would disambiguate beliefs about ‘where the keys are in the world’. Then, they recalled that they had recently changed the key fob—a fob that used to be yellow was now bright pink (a souvenir from a Pet Shop Boys tour gig). The keys, previously invisible, immediately jumped out and were discovered. In the terms just introduced, the revised prediction that the fob should be pink altered attention (the precision-weightings on specific kinds of visual sensory information) allowing them to actively search the scene in a new way—a search in which the specific shade of Pet Shop Boys pink enabled especially salient actions to be performed. Taken together, action and perception form the kind of loop characteristic of selective attention [42].

### 3.2. The role of the environment in attention

Change to the environment will often impact salience. For instance, by painting white lines in the middle of the road, we alter the salience of actions (such as visual saccades) directed at that spatial location. Those actions now figure crucially in simple perception–action loops that serve to keep the car on the right side of a winding mountain road. This suggests a third way of understanding attentional phenomena—one that brings enactive embodied and extended perspectives in cognitive archaeology into dialogue with important work on ecological and embodied cognition [22,28,55]. Covert precision weighing and overt selective sampling are mechanisms that can only work for agents embedded in a world that affords a certain degree of structure. For instance, markings on roads are only effective if and when drivers can recognize and agree upon their meaning, acknowledging their special salience for the selection and control of driving actions.

Accordingly, to get a full picture of attentional salience, we need to take into account the way in which action resolves uncertainty about states of the world, that is by considering: (i) the salience of action, and (ii), the precision of the environment in which action is performed. Computationally, the precision of the environment can be modelled as the precision of the parameters of a generative process [26,51,56], which is a description of how world states generate the observations driving action and perceptual dynamics of a generative model. In the case of the white lines on the winding road, the precision of the environment, or generative process would be considered as the visibility of the line. For instance, if lines are covered in snow or sand, they can be said to afford imprecise sensory visual information about where the line might be. In turn, freshly painted lines on a clear day would afford precise sensory information as to where the line is, and as to what driving behaviour is allowed on the section of road. If the precision of the lines is low, the sensory visual information will be patchy and irregular, thereby potentially leading to false perceptual inference (e.g. seeing dotted lines instead of the real full lines) and maladaptive actions (e.g. overtaking a car in a section where the line is full).

Attention and salience responds to, and are driven by environmental precision. For instance, as a response to low environmental precision, covert and overt attentional strategies can be adjusted by internally modulating the gain, or precision afforded to certain sensory inputs (e.g. not paying attention to what the newscaster says on the radio), or simply by overtly selecting relevant sensations (e.g. by simply turning down the radio, leaning forward, squinting the eyes to better see the road, while lowering the speed of the vehicle). The selection of relevant information is equivalent to engaging the salient action, that is, the action that will bring about sensory information that will disambiguate beliefs about the structure of the environment (e.g. looking for a turn ahead, under the assumption that lines are full under hazardous conditions). Indeed, a salient action is one that is believed to entail a change in beliefs about the states of the world (e.g. ‘now I think that the line on the road is full’). In turn, an action that would be less salient would be one that would not entail a change in beliefs (e.g. looking at your watch to see if the line is full).

Precision modulation on environmental parameters, as the counterpart to gain control in the brain (cf. box 1), may be done by clearing the sand or the snow. The difference is that precision modulation, or gain control, does not operate autonomously in the generative process, as is the case for the generative model. Gain control of environmental parameters is realized by explicit and implicit modifications of the environment (e.g. requesting snowplows), which can be performed at the individual or the group level. The example of lines and markings on the road illustrates the fact that the complex interplay between cognition, signs, symbols and artefacts that guarantee human adaptive action in a fluctuating world is constantly tuned through inner, active and environmental mechanisms of information processing that rest on neurobiological, behavioural and material forms of engagement with the world. Based on the concepts of precision modulation, salience of action and precision of the environment, in the next section, we describe a computational framework for modelling and studying the manner in which artefacts may drive the looping interaction between precision weighing, salience and active sampling and environmental precision; we describe CCA of attention.

## 4. Computational cognitive archaeology of attention

### 4.1. A tentative definition of computational cognitivearchaeology

Tentatively, CCA can be defined as ‘the study of past human minds through the computational modelling of the archaeological record’. Its first task would be, accordingly, to describe how the evolution of ‘things’ (i.e. artefacts) might be modelled by adopting a computational framework. This may be viewed as an extension of the work already proposed by Tom Wynn in his 1979 paper [57] on computational, or rather algorithmic operations that underwrite the production of tools in the archaeological records. The extension is that not only should a cognitive computational archaeology of selective attention identify the algorithmic steps that lead to the production of artefacts, but it should also model the dynamics of the behaviour of the artefact *per se* in its making and its potential effects on the cognitive processes of the artefact maker, such as selective attention. As a note, we would like to point out that there is the chance that a pure science of artefacts’ evolution does not, in fact, exist, and that the regularities that we observe are instead rooted in changing social and manufacturing contexts. However, even in this case, the models developed by the CCA would be functional as heuristics, supporting archaeological inferences about minds of the past.

A difficulty with CCA is that, given its starting assumption concerning the embodied, embedded and extended nature of cognition, the computational frameworks used by CCA should be able to reflect the way cognitive phenomena can be mapped onto the materiality of the environment. That is, CCA ought to use a computational method that imports, or extends into the domain of culture cognitive processes that are typically modelled as belonging to humans and animals only. CCA should offer a computational model of the ‘cognitive life of things’, as it were. Such frameworks should allow the mapping of key concepts of cognition and materiality onto concepts applied in computational models. Such a mapping is the one that we proposed in the previous section concerning sensory attention as precision neuromodulation, or gain control, and environmental attention as precision over environmental parameters embodied by artefacts.

The extent to which real changes in the environment can be expected to make sense, under the light of such computational constructs, will of course depend on the extent to which the models of CCA reflect the complexity of social and historical dynamics having shaped the modelled artefacts. Accordingly, it is necessary to be careful with claims according to which it is possible to make inferences about the historical and inner-cognitive context of agents from models of artefacts alone. Rather, the kind of models developed by CCA, including the one proposed in this paper, should be viewed as providing an additional piece to the puzzle of cognitive, archaeological, historical and geographical evidence that archaeologists and cognitive archaeologists routinely deal with [58–60]. ‘Computational’ pieces of evidence about past minds derived from computational studies of the archaeological record should obviously be weighed against all other sources of evidence.

One candidate for CCA that would allow the mapping of cognitive concepts onto material phenomena is the theory of ActInf in theoretical neurobiology introduced above [45,61]. Standard formulations of ActInf use Markovian models to describe the way perception and action unfold as processes of hidden state inference and of predicted sensory observation. In this section, we introduce the general concept of Markovian models such as used in ActInf, and stress the aspects of that class of models that we will reuse in the next section to map cognitive concepts such as attention and gain control onto artefacts, so as to propose hypotheses on how such cognitive functions may have been harnessed by humans of the past in their own cognition.

### 4.2. A candidate model for a computational cognitive archeology of attention

Markovian models can be used to perform statistical forecasts over unknown or ‘hidden’ states of interests that relate to some known outcomes that are generated by those states. Based on observed outcomes (e.g. ‘I was told that it is raining’) and some prior knowledge of what causes observations (e.g. ‘when it is raining, there are usually clouds in the sky’) and some knowledge of the relation between observations and causes, or unknown states (a.k.a. likelihood), one can attempt to forecast the weather (e.g. ‘it must be cloudy and not sunny right now’). The forecast, in Bayesian terms, amounts to inferring the posterior probability of the unknown state (e.g. of the weather). Forecasting can be done over multiple time steps to allow one to predict in the future what unknown states will most probably occur.

**Figure 1.**
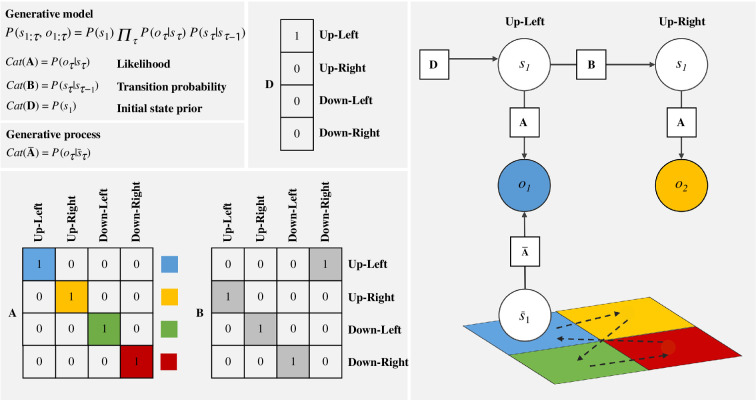
Hidden Markov model (HMM) of cognitive functions in a simple visual foraging task. This schematic presents a Bayesian generative model represented as a HMM that forecasts or infers hidden states (*s*) corresponding to locations in a 2 × 2 grid environment affording different colours. The task of the agent is simply to explore the 2 × 2 grid. The agent is modelled as the HMM. *Left*: formal description of the HMM with states ‘s’ and observations ‘o’. The HMMl corresponds to the joint probability of states and observations, whose relation is captured by the parameters A (i.e. likelihood parameter), B (i.e. transition probability) and D (i.e. initial state parameter), represented as matrices whose columns correspond to categorical distributions (Cat). The parameters in this model include deterministic mappings (all 1 or 0), which assumes that there is no uncertainty in the parameters. *Right*: the visual foraging behaviour afforded by such a model is very simple. The model starts in the location ‘up left’, such as prescribed by the parameter D, and infers the ‘blue’ observation, such as prescribed by the parameter A. The model then moves to the ‘up right’ location and infers that the observation will be yellow, such as prescribed by the parameters A and B. For an application of this broad picture to a simplified archaeological case study, see figure 2.

As applied to cognition, the concept of weather forecasting translates to that of ‘cognition as inference’ over beliefs about how the world unfolds and the sensation it affords, such as discussed in §3. Aligned with our discussion above, a cognitive phenomenon such as attention is viewed as mechanisms modulating the precision of the parameters forming the Markovian generative model, and upon which action and perception unfold. A typical behaviour modelled using the Markovian formulation of ActInf would be the one of visual foraging [43]. In its simplest form, such a model would assume some hidden states that correspond to locations on a grid (e.g. a 2 × 2 grid), that each affords sensory visual information (e.g. different colours) with a given level of probability (e.g. ‘the upper-left position is most probably blue’). The behaviour simulated by such a model would be the gazing of the simulated agent across the different locations (see figure 1).

**Figure 2.**
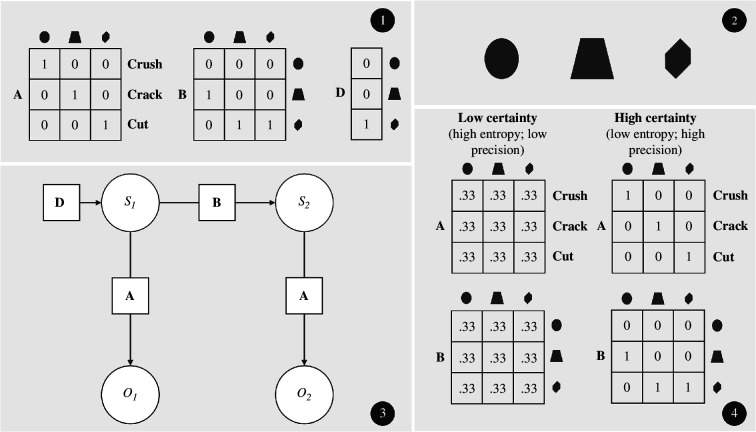
(1) Description of the model parameters of the HMM presented in (3). The A parameter corresponds to all the probability mappings between states (i.e. hammer shapes) and observations (i.e. the hammer’s function). The B parameter corresponds to the state transition probabilities (i.e. mappings between states at time *t* and *t* −1). The D parameter corresponds to the initial state probability of the hammer being in one of its three probable states or shapes. Each column corresponds to a probability distribution, the entropy of which can be summed up with that over the other columns to obtain the environmental entropy, or environmental precision of the entire matrix. (2) Illustration of the meaning of the states. Here, the HMM represents how an object like a hammer and its associated functions may evolve over time, assuming that different shapes call upon different functions, and that shape will change over time relative to the function engaged by the users. (3) A formal description of the evolution of a hammer using a HMM. (4) The right panel presents an A and a B parameter with high precision. All the mappings are deterministic (1 or 0) such that every time that a function (e.g. cutting) will be observed, it will mean that that function will have been caused by a sharp tool, at 100%. Such a high precision in the B parameter means that if we find a certain typical shape at a given site (e.g. rounded shape hammers) for a given archaeological period, we are certain that we will find artefacts mostly with the next shape (e.g. squared hammer) in the next period. The opposite applies to the left panel, which presents the highest level of uncertainty. This would assume that in the archaeological record, shapes rarely match with the function, and that shapes rarely remain the same, nor evolve in a directed fashion (e.g. from round to square to sharp).

Action, in such a simple model for visual foraging, would refer to the moving of the eyes across locations, understood as the inference of where the agent currently is and where it will be next. Sensory perception would, instead, amount to the inference of the colour afforded by the current and future location (e.g. I see ‘blue’ and as I move to the right I am seeing ‘yellow’, see figure 1). Action heavily depends on the agent’s beliefs about the probability of transitioning (i.e. transition parameter B in figure 1) between locations in the grid (e.g. the agent may tend to read from left to right, and thus tend to move from upper left to upper right). In turn, perception heavily depends on the agent’s beliefs about the sensations in different locations (a.k.a. likelihood parameter A in figure 1).

In such a model, what we called ‘sensory’ attention above amounts to the uncertainty, or Shannon entropy over model parameters (e.g. A and B), the modulation over which would correspond to gain control. In such a setting, attention allows the agent to weigh sensory evidence to form a more accurate perception of the state of the environment (e.g. the true distribution of colours on the grid). Crucially, all those inferences would be based on sensory observations whose probability of occurring depend on the true state of the world, or generative process (A_bar in figure 1). For instance, if a visual foraging task is performed over a visual setting that reflects light in an irregular fashion, this may create uncertainty over the probability of colours being afforded at the different locations, thereby biasing the agent towards misperceptions. Returning to the example of the white lines on the road, which here would represent the ‘A_bar’ parameters, low visibility caused by snow or sand would be modelled as high entropy, or low certainty over the probability of the environment affording certain sensory visual information. Accordingly, environmental ‘attention’ understood as environmental precision corresponds to the uncertainty in the environmental parameter of the generative process, which biases cognitive functions such as perception (i.e. A_bar, figure 1).

In the remainder of this article, we explore the way artefacts can be modelled using generative models endowed with parameters affording different levels of uncertainty, thereby guiding cognitive functions such as action and perception in different ways. Our starting assumption is that, in the same way, one can model human behaviour as a process of hidden state inference and of outcome predictions unfolding over time based on sensory observations, it is also possible to model artefacts’ evolution, considered as hidden state inference and outcome prediction unfolding overtime. Whereas hidden states of the brain represent true states of the world, and observations represent sensory information afforded by those true states, we assume that hidden states of artefacts represent material features (e.g. geometries) of artefacts, as they evolve over time as well as the type of actions, or functions that such geometries afford [62]. Indeed, instead of modelling with a hidden Markov model (HMM) the sensory causes and sensory observations of a brain whose inference underwrites perception and action, we will adopt a HMM to model the material features and the functions of artefacts that underwrite the way artefacts shape and use unfold over developmental and cultural evolutionary time, and how these feature drive cognitive functions such as attention.

## 5. Knapping and a computational archaeology of attention

In the previous section, we tentatively defined CCA as the study of past human minds through the computational modelling of the archaeological record. We proposed that one could use a HMM, namely under the theory of ActInf, to model the way artefacts evolve and change overtime as a function of the way artefacts are used. This section explores how, indeed, model artefacts can be modelled as HMMs, and crucially, how modelling artefacts in such a way may give us an insight into how artefacts might have evolved to shape attention, understood as a coordination of sensory precision, salience and environmental precision.

We propose a model of knapping as a form of technology whose material component may be mapped onto HMMs under ActInf. The evolution of knapping can be characterized by key socio-cognitive functions such as material production, social collaboration and social reproduction. The goal of the computational approach sketched in this section will be to illustrate how modelling the materiality of knapping could inform on the way such materiality may have contributed to the performance of the socio-cognitive functions of knapping. Within the context of the proposed ActInf approach, we shall sketch a model of the way the environmental precision of the kind of artefacts involved in knapping may support the functions characteristic of knapping technology.

### 5.1. Artefacts, technologies and knapping

*Homo* is the only mammal found across the globe, and it is the one having had the most impact on the planetary biomass [63]. Such a demographic potential despite the high cost of child rearing [64,65] and slow brain maturation [66,67] would have been enabled by biocultural reproductive strategies [68] fostering resources sharing, collaborative foraging and other forms of social conditions afforded by the human cultural niche [69]. Such strategies would have led to feedback cycles between food resource production, population growth, reduced mortality and accelerated reproduction [70] leading to increased life span, cognitive capacities and intergenerational passing of cultural and technical traditions.

Technical traditions, such as the production of tools and artefacts, which can be embodied (e.g. hammer), perceptual (e.g. spectacles), cognitive (e.g. notebook) or affective (e.g. ‘Guernica’ of Pablo Picasso) [71], can be transmitted and cumulated into even more sophisticated technologies [72]. Thus, with technologies, we refer to techniques (e.g. stone fracture and painting), tools (e.g. hammer; brushes), artefacts (e.g. Karari scrapers and Guernica), and knowledge (e.g. motor skills and properties of colours) that form a domain of human activity (e.g. knapping and oil painting) characterized by a set of behaviours (for a distinction between technology and technique, see [73]).

According to Stout [72], the transmission and evolution of technologies depend on three features, which make up the behavioural domain of the technology itself: (i) material production (i.e. technologies are born from a characteristic production pattern), (ii) social collaboration (i.e. technologies allow individuals to align and to coordinate), and (iii) cultural reproduction (i.e. technologies involve the intergenerational transmission of skilled action) (see box 2). Taken together, the three features of the definition of technologies proposed by Stout [72] suggest that a technology suited to cumulative cultural evolution will tend to be: (i) materially produced in a way that guarantees its *transmission*, which involves a *transmissible medium*, and the ability of the technology to afford *cognitive offloading*; (ii) affords modes of social *coordination* fostering collaboration leading to *specialization* and behavioural *synchronization*; (iii) leverages *skilled action* acquired through implicit and explicit processes of *collaborative learning* of predictive cognitive sensory–motor schema.

Box 2. **Key features of transmissible technologies**

*Material production*

The first feature of the behavioural domain of technologies according to Stout [72] is its material production. The material production of a technology is related to three parameters: its complexity, its medium and the cultural transmission of both. Technological complexity is a process of open-ended accumulation of components and procedures [74], characterized by increase in efficiency and effectiveness [75], and as such, the ultimate sign of cumulative cultural evolution. For cumulating, technology and medium need to be transmitted and modified, and this happens in two directions. On the one hand, the technological complexity unfolds into a medium (e.g. paper), which can be re-engaged over time, and can allow collaborative improvement on both the medium and its technology. On the other hand, the technological medium is required to possess the ability to be bequeathed across generations. Additionally, transmissibility, as the durability of a medium (i.e. an artefact) and the extent to which it will be re-engaged by conspecifics, depends on its ability to: (i) externalize and scaffold cognition [22,76,77] and (ii) cue the expected behaviour under the affordances of the object itself [49,50]. That is, a medium is durable as long as it can be the target for the offloading of various social and non-social cognitive functions [51,78], which, in turn, justifies why technological production accounts for low rates of information dropout across generations, even in the absence of complex social learning strategies [79–82]. That is so, we argue, because transmission rests, eventually, on the extent to which the technology will offload cognitive functions, and the extent to which its embodiment will allow it to be effectively passed over generations.

*Social collaboration*

A second feature of the behavioural domain of technologies is the social collaboration it affords. One key feature of tools changing along the continuum going from simple (i.e. a tool) to more complex contemporary technologies is the extent to which the technology affords the coordination of behaviour across multiple individuals [72], as highlighted, for instance, by the specialization process in the production line (e.g. the creation of supply chains improving coordination and workforce productivity). Coordination, or the (social) synchronization of behaviour through (asocial) cues, rests on (i) a tendency of learning about the world, as a combination of objects and people [83] and (ii) the ability of the technology to allow for the prediction of the partner’s behaviour, central to phenomena such as mind reading, shared intention and collective actions [84,85]. Coordination, thus, can be achieved in groups of different sizes (e.g. two individuals coordinating using their watch to arrive on time; pupils and teachers coordinating their actions at the ring of the bell), explaining micro and macro phenomena.

*Cultural reproduction*

The third feature of the behavioural domain of technologies argued by Stout [72] is the cultural reproduction of technologies. Technological learning, beyond operating on transmission and copying, involves collaborative processes of skill reproduction [86,87]. In humans, the material production of technology, and its innovation, lie on high-fidelity transmission of skilled actions, which is achieved through: (i) exploration of, and active engagement with social and asocial [83], and ultimately cultural environments [88] and (ii) throughout social learning channels, particularly imitation [89,90]. Some authors have even argued that technological production can be considered the physical representation of the social channel, as the preferential learning mechanism in the human lineage [91–93]. High-fidelity social transmission requires, primarily, the ability to target expertise (i.e. socially cued observable behaviours, in association with achieved goals, performances and/or socio-normative practices) and experts (i.e. social models taken as masters, eventually responsible for unintentional and intentional instruction and teaching). Skilled expertise and experts guide behaviours (actions and learning), and functions (perception and attention), through acquired [94] predictive cognitive models, necessary for technological production and innovation. That is, expertise and experts, leveraged as exemplars, constrain and guide possible variations in technological production, and bias the probability of adoption and successful copying of technologies [72].

We suggest that a theory of artefacts and of the cognitive functions they afford within the context of a technology (e.g. [95]) should explain how artefacts contribute to the performance of the three features described above. That is, there is something cognitive [71,73] about artefacts that make them suitable for material production, social collaboration and cultural transmission, which a theory of artefacts in cognitive archaeology should address. One ought to be able to frame in cognitive terms the fact that a ‘core stone’ in knapping technology is a ‘continuously available external resource structuring behaviour’ [95], which helps explain the cultural reproduction of knapping from the point of view of its user. Even if not all tools support, or have ‘high’ cognitive functions (e.g. memory and mentalization)—for some tools might simply have a pragmatic function (e.g. hammering [73])—we cannot rule out the hypothesis that, over developmental time, at least some simple tools might scaffold higher cognitive processes. For instance, once the pragmatic function of the hammer has been acquired, ‘hammering’ can turn into an integrative part of the mental model and representation of the process (i.e. a high cognitive function: one must have a sense of how a hammer can be used on a nail to imagine hammering a nail in a wall to hang a picture). This suggests that, over time, higher cognitive functions such as imagination and planning will become embedded within the pragmatic functions afforded by simple artefacts (e.g. hang-ability of the frame on the nail in the wall). The goal of the next section is, then, to suggest how this might happen, through the creation of a map of as many features as possible onto our computational model of artefacts. Additionally, we propose that many of these features are supported by what we termed ‘environmental precision’.

### 5.2. Computational cognitive archaeology of attention in knapping

Knapping is the practice of ‘striking a stone with another stone or something softer than a stone, such as bone or wood’ [15]. Stone fractures range from unintentional, as shown by products left after percussion activities in non-human primates (apes and monkeys) [96–98], to intentional and controlled, aiming at the construction of tools and other artefacts [72,95]. The first tools, the result of intentional knapping, were sharp flakes. Used primarily for processing carcasses [99], they became essential to the survival of early *Homo*, as their global migration patterns and evolution seem to suggest [100]. We focus, here, on knapping as an illustration of a technology that could be modelled using the method highlighted above, for knapping is a technology that presents many of the features argued by Stout [72]. Moreover, knapping is a well-known technology and many readers will be familiar with it. Readers should be aware that our presentation of knapping serves a heuristic purpose. A more in-depth study of knapping tools and the way their structure and evolution may map onto the parameters of a computational model would be required, if any archaeological conclusion is to be drawn from an approach such as the one proposed here.

The question that we now turn to is how one can use the computational construct of environmental precision to help explain how the materiality of knapping (e.g. a ‘knapping tool’) contributed to material production, cultural reproduction and social collaboration, coordination and synchronization in knapping technology. We exploit a (hypothetical) ‘knapping tool’ that we model using the resources of the HMM to inquire on that question. Following the description of the HMM in figure 1 above, the model of a knapping tool that we present in this section is described in terms of the joint probability of a series of hidden states making up the tool, and a series of observations that related to those hidden states (figure 2). Here, heuristically, we assume that hidden states correspond to the possible ‘shapes’ that the knapping tool might have taken throughout human history. Different shapes of knapping tools have been found throughout the archaeological record, such as irregular and sharp flakes detached from a core (e.g. Lokalalei & Kenya [101]), Acheulean bi-faces, symmetrical hand axes, parallelogram-like shaped, with rough edges from Olduvai (Tanzania), 1.7 MY (e.g. [102]), Barranc-de-la-Boella’s pointy edge and almost unworked round core (e.g. [103]), and more refined such tools found in the Israel area (e.g. [104]), round hammerstones found in China, slightly levelled on the side of use (e.g. [105]). In the context of the HMM presented in this article, for illustrative purposes, we make the simple assumption that tools can take one of three possible geometrical shapes, namely, ‘rounded’, ‘squared’ or ‘sharp’. Under the assumption that the process having led to the current distribution in the archaeological record is random at the scale of human populations, it is reasonable to assume that the probability of a given knapping tool to take one out of those possible shapes would depend on their frequency in the archaeological record (e.g. if rounded knapping tools have been found at one site more often than squared ones, we assume that most of the knapping tools were, probably, rounded). Those frequencies, referring to figure 1, could be used to parameterize the ‘transition’ parameter B of the HMM, which here would report the probability of a typical knapping tool to take one of the possible shapes over time.

The HMM such as described in figure 1 also includes a ‘likelihood’ parameter A that maps the probabilities of certain observations (e.g. the colours in figure 1) onto the possible states that the system of interest could be in (e.g. the locations in figure 1). Here, the states of interest are the shapes, and the system is the knapping tool. Accordingly, we suggest that the ‘observations’ of the knapping-tool system correspond to the kind of actions, which can be performed with the knapping tool, by a *Homo*-like animal. Here, the assumption is that the geometry of the shapes (e.g. rounded shape) cause, as it were, possible actions that the knapping-tool system affords. For instance, a rounded shape might tend to cause people to use the tools for crushing actions, a squared shape might afford cracking actions and a sharp tool, cutting actions. We thus use crushing, cracking and cutting as the ‘observations’ of the knapping-tool system implemented by the HMM. These observations map onto corresponding shapes, or states. One can imagine the parameterization of the mapping between actions (i.e. observations) and shapes (i.e. states) to be done based on the sort of remains that are typically found around tools with different shapes. For instance, if animal bones with cut marks are found more often around sharp than around rounded tools, this suggests that the mapping of cutting action onto the sharp shape should have a greater probability than the mapping between the cutting action and the rounded shape.

Note that shapes and actions are specific to the kind of knapping-tool system that we are modelling. If one were to model a different tool or artefact with the proposed method, one would likely use a different set of states of observations. For instance, in modelling something like a pictogram, the states of the pictogram may be geometric forms such as lines (horizontal and vertical), or more sophisticated shapes such as triangles and circles, or even more semantically heavy graphics, such as characters or animals. These ‘pictographic’ states would then be mapped onto observations that could be actions such as gazing direction (e.g. looking up) or ‘not sitting’. Or closer to our purpose here, one could model an artefact like a stone core. In the following discussion, we are assuming a very generic knapping tool that would take different shapes, and so, we are not specifying what kind of tool the knapping tool is, other than a tool that ‘changes shapes and functions’ and that ‘could have been used in knapping’.

The advantage of using the proposed modelling technique is that it discovers the probabilistic structure of the evolution of the modelled artefact, in this case, our knapping tool. Practically, this can be done by updating the parameters A and B by using a simple method of count, wherein each piece of evidence of a mapping such as expressed in either of the matrices A or B increases the probability of that mapping in the corresponding cell location by, for instance, +1 (e.g. if the mapping 0.3, then it becomes 1.3). When normalizing the matrix to ensure that each distribution sums to 1, the cell with the added count will proportionally increase in probability compared with its competing mappings (e.g. 1.3 will become 0.65 if the other element in the distribution was 0.7, which will then become 0.35). Importantly, nothing restricts the update of the parameters based on observed evidence to strictly empirical evidence. Any evidence deemed worthy of consideration can be added to the model, whether it comes from the archaeological record, or from conclusions of well-supported theories. This further allows to frame the history of the artefact in terms of the computational construct of environmental precision, which helps explain the way the artefact has evolved to drive the cognitive function of attention. Recall that in computational cognitive neuroscience, attention can be viewed as a process that brings together internal, behavioural and external components, each one of them feeding on another, to guarantee adaptive actions. By modelling artefacts as HMMs with A and B parameters that can each afford a different degree of environment precision (see figure 2), one can better understand how that artefact might have evolved, driving along human attention. Crucially for our purpose here, this strategy further allows us to account for the socio-cognitive functions of the material counterpart of technologies.

Returning to the modelling of artefacts involved in knapping technology more specifically, knapping might involve the use of a stone, handled and controlled with the function of a ‘hammer’, to detach flakes from another stone, manipulated with the function of a ‘core’. The *material production* of the final tool (e.g. a ‘cutting’ flake) involves the employment of an embodied tool [71], the hammer: the stone-hammer is leveraged as an (external) guide to sensory–motor loops, the precision and strength of which are provoked by and scaffolded within the hammer itself. In terms of *cultural reproduction*, the acquisition of the skills required for controlled stone fracture involves the guided learning of cognitive models that allow for precise body kinematics and positioning [106]. This requires deliberate practice, supported through a structured learning environment, in association with apprenticeship instruction, guided learning, or intentional and explicit teaching [107]. In any case, knapping learning leverages *social collaboration*, *coordination* and *synchronization*, which are required to optimize knapping technology over intergenerational timescales [108]. Next, we highlight how the construct of environmental precision in cognitive computational archaeology may help describe how hammers, understood as the material counterpart of knapping technology, may have contributed to the material production, cultural reproduction, and social collaborative functions of knapping technology.

### 5.3. What the model affords

The precision or entropy over the likelihood parameter A understood as environmental precision captures the probability of certain shapes (e.g. round, square and sharp) co-occurring, say, in the archaeological record, with functions (e.g. crushing, cracking or cutting). Such likelihood parameters affording high precision when trained on archaeological data would suggest that the population who generated the record, might have achieved a high level of efficiency (i.e. reliably engaging a function under a specific shape instead of attempting various functions across multiple different shapes), suggesting also certain levels of social coordination—as specific shapes would be reliably used for specific functions during knapping. Variability in shape–function mappings in the archaeological record would still suggest lower precision, and thereby specialized, efficient and skilled action (e.g. if all the possible versions of the tool’s shapes were used for all the possible functions).

Second, the precision, or entropy over the transition probability matrix B that maps the probability of transitioning between artefacts physical features over time, reflects the extent to which artefacts’ shapes remain constant in the archaeological record, for a specific class of artefacts (e.g. squared version of the hammer remaining square across time), and therefore speaks for the material reproduction of the technology using that artefact. Accordingly, robust material reproduction can be expected to be enabled by reliable transition probabilities in the B parameter. Crucially, an archaeological record affording high precision for both, the transition matrix and the likelihood matrix A, would be evidence for both, transmissibility of skilled action and specialization, as geometries, or shapes reliably affording certain functions (e.g. crushing) would have been reliably passed over generations (e.g. a hammer with sharp geometry reliably affording cutting having a high probability of remaining sharp in the next generation, and therefore of affording cutting).

Another interesting computational feature of the HMM presented in figure 2 is that changes in the probability mappings of transition, and the likelihood, can alone provide information on the evolution of the technology. For instance, a transition could encode the vector of evolution of an artefact’s shape (e.g. is there a probability that the artefact moves from round to square, but not from square back to round?). In the archaeological records, we can find evidence of transitions from multipurpose round-shaped hammers to tear-drop hand axes. Some of early hand axes show an almost unworked, round core, as in Barranc-de-la-Boella (Spain) [103], which appear to have been refined into chisel-like shape (cf. the Israeli case in [104]). Interestingly, the probability of transitioning among possible artefacts’ shapes may also give indications about phenomena, such as loss of skills, specialization and knowledge (e.g. if reliably transmitted, low entropy shapes and functions parameters become suddenly uncertain). Stone flaking was started by hominins around 3.3 Ma (Lomekwian, Kenya), and mastered from around 2.6 Ma (Oldowan, Ethiopia). Then it was followed by the appearance of tear-drop-shaped handaxes (i.e. bifaces) around 1.75 Ma and reduction techniques in the production of stone-tools around 1.6 Ma. For cognitive archaeologists, this progression and the persistence of stone tools provide robust evidence for the evolution of cognitive abilities of early human species [109,110]. The inference to ground that claim is done in one of two ways. First, tool types or shapes are thought to reflect the geometrical design that was intended by the toolmaker. The more regular a design is, the higher the cognitive abilities required to produce the tool are assumed to be. And so, if a transition from irregular to regular designs is observed in the archaeological record, one assumes that a cognitive transition might have happened [111]. Second, one can assume that the technical sophistication of the tool itself may reflect the cognitive abilities of the toolmaker. In this case, the inference is made based on empirical evidence coming from studies with contemporary expert knappers (e.g. brain-imaging studies [108] and body kinesthetics [112]). Evidence from both sources can be combined to draw more robust conclusions. Yet, this remains only half of the puzzle, as those two sources of evidence speak to only one direction of fit in the relation between tools, artefacts and artefact or toolmakers; the direction that goes from the maker’s brain to the object. This direction of fit assumes that ‘some pre-formulated “idea” or “mental representation” [...] inside our head precedes and causes the materialization of the [tool or artefact] in the outside world’ [113]. From an embodied perspective, the other direction of fit, the one that goes from the tool to the artefact maker’s brain also matters. The model on offer explores that second direction of fit. Specifically, it is designed to provide information on the way features of the artefact contribute to its own transformation (geometrical or else, depending on what features are being modelled), in relation to the way those features have been engaged by the artefact or toolmaker. For instance, there is evidence that the acquisition of Palaeolithic tool-making skills engages recently evolved brain regions involved in higher cognitive functions such as planning and language [114]. This line of evidence should be combined with evidence about how features of the artefact make actions characteristic of higher cognitive function possible. Indeed, the assumption of the embodied approach to cognitive archaeology is that there is something about the materiality of objects that makes them such that cognizing in a certain way becomes possible when engaging objects. On that view, one should seek to combine evidence coming from cognitive neuroscience with evidence that tools and artefacts’ evolution renders certain actions possible (e.g. cut-ability, crush-ability, flaking-ability, etc.). This is what inferring and simulating trajectories of tool evolution with a model such as a HMM would allow. A HMM like that presented in figure 2 allows us to infer what future actions are to be expected as the tool changes shape. If after training the model with observations from the archaeological record or other trustworthy piece of evidence, one finds that the actions that are expected are the actions that are conducive to higher cognitive abilities such as planning and language, then one can close the ‘evidentiary loop’, by connecting evidence that ‘certain cognitive functions are involved in making certain tools’ and evidence that ‘certain tools afford certain action that is representative of, or conducive to certain cognitive functions’.

As a final observation, one should note that from a computational point of view, environmental precision might have an impact on learning in other domains of attention involved in a technology. Namely, those domains that belong to the user *per se*, such as sensory precision and attentional salience. It is so, we argue, because they are also precision, or uncertainty driven, and they can be learned over time (e.g. learning to attend to certain sensory entries that will therefore become relevant). In a context where environmental uncertainty is high, one should expect the learning of parameters yielding an equally high level of uncertainty, and therefore, of limited abilities to accomplish task invited by the artefacts affording low certainty (e.g. knapping with a hammer that mixes awkwardly round, square and sharp shapes and mediocre at all the functions). Accordingly, the strategy proposed in this article may allow us to take a dive into the study of the attentional style [115], which was characteristic of the population, whose artefacts were used to train the HMM. That said, it may be argued that our proposed HMM does nothing over and above what archaeologists already do when they make an inference from the archaeological record to cognition of humans’ ancestors. There is a sense in which this is correct, especially when considering the kind of model on offer, which is derived from cognitive neuroscience, and is aimed at simulating the behaviour of neural and non-neural systems such as those of archaeologists’ brains and objects of inquiry. But even if this were true, there would still be an advantage to using a computational model to perform the inference. For instance, a computational model will provide metrics that speak to how good the inference is, or about how certain we should be about the model that produces the inference. While two archaeologists can agree on the goodness of their inference—this is the very logic of a scientific community of practice—being able to do so based in part on objective metrics offered by computational modelling would certainly be an advantage.

## 6. Computational cognitive archeology: wherever next

This article proposed a computational approach to the cognitive archaeology of attention. We explored three constructs related to attention in theoretical neurobiology; the construct of attention as gain control, or precision of neurobiologically encoded beliefs, the construct of salience as an attribute of action or potential for information gain, and the construct of environmental precision understood as the precision of environmental parameters. We then reused the computational construct of environmental precision to illustrate how artefacts within the context of a technology can be modelled as devices that steer attention so as to support the key features of technology; (i) material production (i.e. technologies are born from a characteristic production pattern), (ii) social collaboration (i.e. technologies allow individuals to align and to coordinate), and (iii) cultural reproduction.

The motivation for such a computational approach to cognitive archaeology, CCA, is to contribute to the variety of evidence that cognitive archaeologists can appeal to in their attempt at inferring past minds based on the archaeological record. The challenge of archaeology is to bring evidence to help climb the ladder of inference. As a historical science, archaeology is not only in the game of acquiring empirical evidence for or against archaeological hypotheses. In archaeology, theories are also evidenced through the accumulation of various sources of evidence [116], from empirical studies to novel archaeological findings. In this context, the role of CCA is to add to the different sources of evidence by providing computer simulation informed by archaeological and cognitive neuroscientific knowledge. In the case of knapping technology that interested us in this paper, the modelled offered—had this modelled been trained on archaeological data, which is possible with the proposed model—could inform us as to the kind of effects on attentional gain and salience the knapping tool may have had on its users in the past, and accordingly, could inform us on the kind of behaviour and styles of material engagements may have occurred in the past (e.g. high precision on function–shape mappings may witness the achievement of a greater level of skilled engagement with the knapping tool).

Important questions remain concerning the scope and limits of the kind of approach we have sketched. For example, there is a clear evolution of what might be thought of as ‘artistic styles’, such as from Renaissance to Baroque in western Europe. Could that kind of change also be explained by the model? To some degree perhaps. We have to be careful, however, not to expect a model like ours to explain too much. Instead, historical change will always reflect a balance of explanatory forces, some of which turn on model-external contingencies such as new trade routes, natural events, new manufacturing capacities and the way populations with different agendas and skill sets are moved around by wars and immigration.

Rather than aiming to capture the full shape of the historical record, the view that we have sketched in this article rests only on the assumption that artefacts, and their production and reproduction play a pivotal role in the construction of mental models, and in triggering and scaffolding cognitive processes and cultural practices. With respect to this specific assumption, follow-up studies could be applied to the investigation of social learning mechanisms, particularly imitation, as we assume that high fidelity is the consequence of material reproduction and technology, and not its cause. In a chimpanzee-like environment, and in chimpanzee-like individuals, even if high fidelity is possible [117,118], it is unlikely to be a solution, albeit latent [119,120]. The reason might well be that it is exactly the massive production of artefacts, typical of genus *Homo*, which scaffold, trigger and make ubiquitous the performance of high fidelity, in tandem with innovation [90]. Advanced technologies eclipse the finality of the actions to the agent (in terms of both, function and shape of the final artefact, which turn ‘opaque’), and the agent needs to rely on both, social and asocial channels contemporarily. That is, for high-fidelity transmission and innovation of technological complexity, all the three pieces of the puzzle (namely, the agent-learner, the expert, and the expertise) need to be contemporarily integrated. Ultimately, even in cases in which the object *alone* also represent the social interaction (as a complex object, for instance, a smartphone, is clearly result of a complex socio-technical structure), or the social interaction refers *implicitly* to an ability or a procedure (as an expert is leveraged as such, because able to do something), the three elements form a quite evident case of extended mind.

Other follow-up studies could be possible in primate archaeology [96]. There, while the model proposes a theory for the evolution of technologies, it could also be usefully applied as a disambiguation tool. For instance, the model could be trained on different ‘flakes’, results of both, non-human primates’ knapping and early *Homo*, and then adopted for proposing a theory of their difference on the basis of, to say, their functionality. Since in this case the model would be trained on real archaeological data, we could also validate its accuracy, in terms of proximity of its results to what is actually found in the archaeological records. On the other hand, if demonstrated to be accurate, the model could eventually be applied in situations where evidence is lacking or in order to fill gaps within the archaeological records. For instance, remains found in a certain site, evident results of tool activities but without the presence of tools, could be used for ‘guessing’ which shape the artefacts could have had—a sort of *Xantopan morgani predicta* in the evolution of technology.

Another possible exploitation of the model is in association with empirical data. For instance, one could study agents’ attention during knapping procedures, referable to various toolmaking industries (adopting eye-tracking techniques). Within an ActInf paradigm, the precision of the beliefs of the agent determines the agent’s attentional level toward social or asocial sensory stimuli, according to the task at hand. In turn, the task itself (its material components) will be more or less precise, such that those material components will afford possibilities for action that will vary in their actionability (e.g. drinkability is precisely afforded by the shape of a mug, whereas it is only imprecisely afforded by the shape of a bowl). Our educated guess is that, in case of ‘simpler’ artefacts, such as those produced in the research of Tennie and colleagues [81,82], the attention stays focused on the materials (particularly, on the stone-core), until the moment in which the final tool (flake) is produced. In more complex industries, such as the production of symmetrical shaped artefacts, as those of the Acheulean period, the focus should shift back and forth, from the stone-core to the model (which, for the reasons we sketched above, could be both a ‘master toolmaker’, but also simply a model artefact), requiring also a more precise use of the material itself (e.g. the power of the stone-hammer has to be modulated more efficiently).

Finally, oftentimes, the functionality of decorative elements found in archaeological records is hard to infer. For instance, in the East Frisian area in the north of Germany, some objects (Muschelgrusgemagerte, i.e. pottery realized with shell-tempered grus) dated roundabout ninth century A.D. present ‘aesthetic elements’ realized with fingerprints and fingertips. Archaeologists that specialize in these archaeological findings have difficulties understanding what those decorative elements are for. Some think they might have been decorations, some read them as signatures of the household thatmade them, some others interpret them as signs of functionality. The reason for such a debate is that these elements do not present the classical structure of decorations. They do not run as a regular pattern on the surface of the objects, but they appear irregularly on the artefacts, and they appear and disappear within the archaeological record. Our model, comparing possibilities of functions and shapes, and trained on other cases in which similar elements were found in other parts of the world and other times, could propose a connection between them and possible functions. A similar problem applies to the inference over the meaning of various artefacts, such as pots and their decorative features. The proposed CCA could fill in the gap in expertise by combining various similar, though geographically distinct archaeological records.

## Data Availability

This article has no additional data.
